# MicroRNA-155 influences B-cell function through PU.1 in rheumatoid arthritis

**DOI:** 10.1038/ncomms12970

**Published:** 2016-09-27

**Authors:** Stefano Alivernini, Mariola Kurowska-Stolarska, Barbara Tolusso, Roberta Benvenuto, Aziza Elmesmari, Silvia Canestri, Luca Petricca, Antonella Mangoni, Anna Laura Fedele, Clara Di Mario, Maria Rita Gigante, Elisa Gremese, Iain B. McInnes, Gianfranco Ferraccioli

**Affiliations:** 1 Division of Rheumatology, Fondazione Policlinico Universitario A. Gemelli, Catholic University of the Sacred Heart, Rome 00168, Italy; 2 Institute of Infection, Immunity and Inflammation, College of Medicine, Veterinary and Life Sciences, University of Glasgow, Glasgow G12 8QQ, UK; 3 Division of Pathology, Fondazione Policlinico Universitario A. Gemelli, Catholic University of the Sacred Heart, Rome 00168, Italy

## Abstract

MicroRNA-155 (miR-155) is an important regulator of B cells in mice. B cells have a critical role in the pathogenesis of rheumatoid arthritis (RA). Here we show that miR-155 is highly expressed in peripheral blood B cells from RA patients compared with healthy individuals, particularly in the IgD^-^CD27^-^ memory B-cell population in ACPA^+^ RA. MiR-155 is highly expressed in RA B cells from patients with synovial tissue containing ectopic germinal centres compared with diffuse synovial tissue. MiR-155 expression is associated reciprocally with lower expression of PU.1 at B-cell level in the synovial compartment. Stimulation of healthy donor B cells with CD40L, anti-IgM, IL-21, CpG, IFN-α, IL-6 or BAFF induces miR-155 and decreases PU.1 expression. Finally, inhibition of endogenous miR-155 in B cells of RA patients restores PU.1 and reduces production of antibodies. Our data suggest that miR-155 is an important regulator of B-cell activation in RA.

## Abstract (web summary)

MiR-155 is thought to inhibit PU.1 and thereby drive antigen-induced B-cell maturation. Here the authors show that patients with rheumatoid arthritis have high B-cell miR-155 expression and that an antagomir can rescue PU.1 expression, suggesting potential therapeutic avenues to treat rheumatoid arthritis.

## Graphical abstract

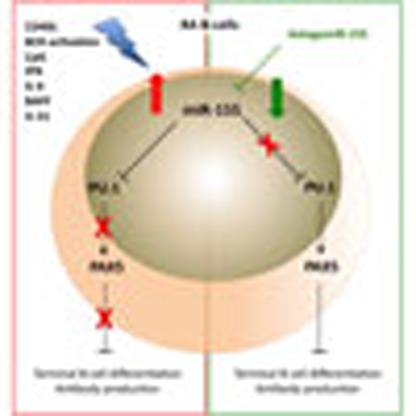

Rheumatoid arthritis (RA) is a chronic inflammatory polyarthritis characterized by clinical and synovial heterogeneity^1^. Histological analysis of RA joints shows that inflammatory cells within the synovial tissue can establish microstructures resembling the follicular structures normally residing in lymphoid organs. The presence of those structures is correlated with compartmentalized accumulation of B and T cells and with a specific cytokine pattern within the synovium^2^. B-cell analysis suggests that these structures function as germinal centres (GC) wherein antigen-activated B cells locally differentiate into effector cells^3^. Moreover, aggregate units are surrounded by anti-citrullinated peptide antibody (ACPA)-producing plasma cells and probably contribute to autoimmune disease progression via activation-induced cytidine deaminase^3^.

Several lines of evidence suggest an important role for specific miRNAs in RA^4^,^5^. MicroRNA-155 (miR-155) has a crucial role in the development of experimental arthritis^6^; previous studies show that miR-155 mutant mice display defective B- and T-cell immunity and abnormal function of antigen presenting cells^7^,^8^. A reduced number of GC B cells are observed in miR-155 deficient mice, whereas miR-155 overexpression has the opposite phenotype^8^. Microarray analysis of B cells activated under conditions that promote class switching to IgG1 suggests that miR-155 regulates expression of many genes, a substantial proportion of which are predicted to be direct targets of miR-155. One of these is the transcription factor PU.1 that is highly expressed in miR-155-deficient B cells. PU.1 overexpression in wild-type B cells results in reduced numbers of antigen-specific IgG1-producing cells indicating that miR-155, through the negative regulation of PU.1 has an important role in antigen-driven B-cell maturation in mice^9^. However, the role of miR-155 in B cells of RA patients has not been described. In particular, understanding epigenetic regulatory mechanisms in RA B cells could facilitate the development of new biomarkers or therapeutic strategies to manage RA.

The aims of our study were therefore: (i) to assess the expression of miR-155 in B cells of RA patients in multiple biological compartments (PB, SF and synovial tissue, respectively), (ii) to evaluate the possible association between miR-155 expression and B-cells activation features (defined as ACPA positivity and ectopic synovial GC frequency), (iii) to assess the relationship between miR-155 and its target PU.1 in synovial tissue and circulating B cells of RA patients and (iv) to investigate the impact of miR-155 on RA B-cell function.

## Results

### Follicles are present in early and long-standing RA

**Figure 1:**
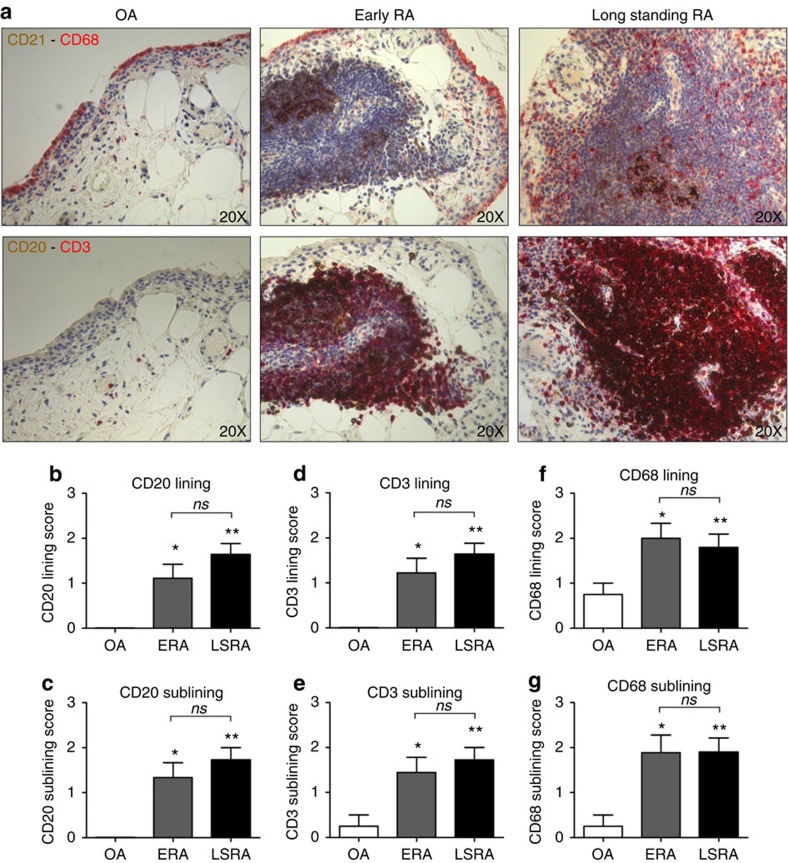
CD20, CD3, CD68 and CD21 in synovial tissue from early and long-standing RA. (**a**) Representative pictures of CD68 (red)/CD21 (DAB, brown) and CD20 (DAB, brown)/CD3 (red) in synovial tissue from early, long-standing RA and OA patients (original magnification, × 20); (**b**-**c**) CD20 staining score for lining and sublining areas of synovial tissue from early (*n*=27) and long-standing RA (*n*=33) patients; * CD20 staining in early RA versus OA (*n*=14) patients; ** CD20 staining in long-standing RA versus OA patients; (**d**–**e**) CD3 staining score for lining and sublining areas in synovial tissue from early (*n*=27) and long-standing RA (*n*=33) patients; * CD3 staining in early RA versus OA patients; ** CD3 staining in long-standing RA versus OA (*n*=14) patients; (**f-g**) CD68 staining score for lining and sublining areas in synovial tissue from early (*n*=27) and long-standing RA (*n*=33) patients; * CD68 staining in early RA versus OA (*n*=14) patients; ** CD68 staining in long-standing RA versus OA patients. Data are mean±s.d., *P≤0.05, **P≤0.01; Mann-Whitney *U*-test; ns=not significant.

Seventy-four patients (60 RA and 14 OA respectively) underwent ultrasound guided synovial tissue biopsy. Demographic and clinical characteristics of enrolled patients subgroups are summarized in Table 1. RA patients were younger (55.9±13.9 years) and had higher systemic inflammation (erythrocyte sedimentation rate (ESR): 45.5±32.8 mm/1st hour) compared to OA patients (age: 64.0±7.3 years, *P*=0.04 and ESR: 26.1±20.6 mm/1st hour, *P*=0.02, Mann-Whitney *U*-test). OA patients were neither ACPA positive nor exhibited synovial follicular structures (Table 1). In contrast, 48.3% of RA patients exhibited synovial follicular structures at synovial tissue biopsy. RA patients were categorized according to disease duration as early RA (*n*=27) or long-standing RA (*n*=33). ERA and LSRA patients show significantly higher CD20^+^, CD3^+^ and CD68^+^ cells infiltration within synovial tissue compared to OA patients (Fig. 1a-g), in the lining and in the sublining areas. No difference in the frequency of ectopic lymphoid structures within synovial tissue was observed between ERA and LSRA patients as evaluated by CD21 staining (14(51.8%) of ERA had synovial follicular pattern versus 15(45.5%) of LSRA had synovial follicular pattern respectively; *P*=0.62, *χ*^2^ test) (Fig. 1a) and (Table 1)). Moreover, we found that CD20, CD3 and CD68 staining scores did not differ between ERA and LSRA patients (Fig. 1a-g).

### miR-155 is highly expressed in RA B cells

Next, we divided RA patients according to seropositivity for ACPA but independent of disease duration. ACPA-positive RA patients exhibited higher frequency of follicular structures within synovial tissue compared to ACPA-negative patients. Twenty-two (68.7%) ACPA-positive RA patients had synovial follicular pattern versus 12 (49.0%) ACPA-negative RA patients with a synovial follicular pattern (*P*=0.04). CD20^+^ cells were overrepresented in the sublining of synovial tissue of ACPA-positive RA patients compared to ACPA-negative patients (*P*=0.01, Mann-Whitney *U*-test) (Supplementary Fig. 1a,b). Among ACPA-positive RA patients we found a direct correlation between ACPA plasma titre and synovial aggregate grade via the histological evaluation [*r*=0.382; *P*=0.01; 95%CI (0.12–0.61), Spearman Rank Correlation coefficient].

**Figure 2:**
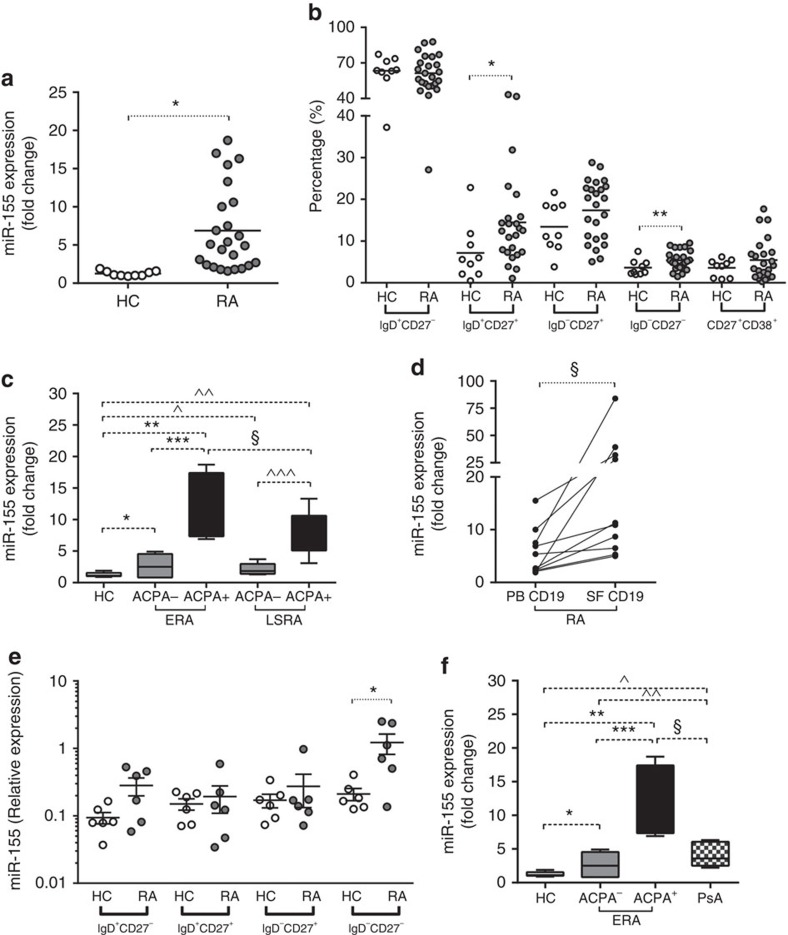
miR-155 expression in CD19^+^ cells from RA, PsA and HC. (**a**) miR-155 expression in peripheral blood (PB) CD19^+^ cells isolated from RA (*n*=24) and HC (*n*=9); *P<0.0001 RA versus HC; Mann-Whitney *U*-test; (**b**) FACS analysis of B-cell subpopulations in PB of RA (*n*=24) and HC (*n*=9); *P=0.02 and **P=0.05 RA versus HC; Mann-Whitney *U*-test; (**c**) miR-155 expression in CD19^+^ cells isolated from PB of RA patients based on disease duration and ACPA positivity; * *P*=0.032 ACPA^-^ ERA versus HC; **P=0.0004 ACPA^+^ ERA versus HC and ***P=0.002 ACPA^+^ ERA versus ACPA^-^ ERA; ^*P*=0.04 ACPA^-^ LSRA versus HC; ^^*P*=0.0002 ACPA^+^ LSRA versus HC and ^^^*P*=0.002 ACPA^+^ LSRA versus ACPA^-^ LSRA; § *P*=0.04 ACPA^+^ ERA versus ACPA^+^ LSRA; Data are shown as mean±s.d., 25–75 percentiles; Mann-Whitney *U*-test; (**d**) miR-155 expression in paired samples (*n*=10) of CD19^+^ cells isolated from PB and synovial fluid (SF) of RA patients; *P=0.04 SF CD19^+^ cells versus PB CD19^+^ cells; Wilcoxon test; (**e**) miR-155 expression in sorted B-cell subpopulations from PB of RA patients (*n*=6) and HC (*n*=6) in three independent experiments. *P=0.02; Mann-Whitney *U*-test; (**f**) miR-155 expression in PB-derived CD19^+^ cells of RA and PsA patients; * *P*=0.032 ACPA^-^ ERA versus HC; **P=0.0004 ACPA^+^ ERA versus HC and ***P=0.002 ACPA^+^ ERA versus ACPA^-^ ERA; ^*P*=PsA versus HC; ^^*P*=0.25 ACPA^-^ ERA versus PsA; §*P*=0.01 ACPA^+^ ERA versus PsA; data are shown as mean±s.d., 25–75 percentiles; Mann-Whitney *U*-test.

We next assessed miR-155 expression in B cells from patients with different clinical features and from different tissue compartments. MiR-155 is expressed at higher levels in RA CD19^+^ cells (*n*=24) compared to CD19^+^ cells isolated from healthy controls (HC) (*n*=9) (*P*<0.0001, Mann-Whitney *U*-test) (Fig. 2a). Flow cytometric analysis (FACS) of CD19^+^ cells shows a higher proportion of pre-switched memory (IgD^+^CD27^+^) (*P*=0.02) and double negative memory B cells (IgD^−^CD27^−^) (*P*=0.05, Mann-Whitney *U*-test) in RA compared to HC (Fig. 2b). Stratification of RA patients according to ACPA positivity revealed that CD19^+^ cells isolated from PB of ACPA positive show higher miR-155 expression than HC and ACPA-negative RA patients (*P*=0.002, Mann-Whitney *U*-test). Moreover, CD19^+^ cells isolated from peripheral blood (PB) of ACPA-positive early RA patients show higher expression of miR-155 then ACPA-positive LSRA patients (*P*=0.002, Mann-Whitney *U*-test) (Fig. 2c). Furthermore, CD19^+^ cells isolated from synovial fluid (SF) exhibit significantly higher miR-155 expression than CD19^+^ cells isolated from paired PB of RA patients (*P*=0.04, Wilcoxon-test) (Fig. 2d). FACS analysis of CD19^+^ cells subpopulations in paired PB-SF samples of RA patients shows that SF compartment is characterized by significantly lower proportion of naive B cells (IgD^+^CD27^-^) (*P*<0.001) and higher proportion of post-switched memory (IgD^-^CD27^+^) (*P*=0.002, Mann-Whitney *U*-test) and double negative memory B cells (IgD^-^CD27^-^) (*P*=0.01, Mann-Whitney *U*-test) compared to the PB compartment (Supplementary Fig. 2). MiR-155 expression in SF-derived CD19^+^ cells directly correlates with double negative memory B cells percentages (IgD^-^CD27^-^) [*r*=0.627; *P*=0.05; 95%CI (0.17–0.95); Spearman Rank Correlation coefficient] suggesting a tight biological link between miR-155 and activated and maturation status of B cells in synovial compartment.

To dissect formally the expression pattern of miR-155 in B-cell subpopulations, we investigated miR-155 expression in sorted HC and ACPA^+^ RA PB CD19^+^ cells and observed that double negative memory RA B cells (IgD^-^CD27^-^) show the highest expression of miR-155 compared to other B-cell subpopulations (Fig. 2e), suggesting that an increased level of miR-155 in RA PB and SF CD19^+^ cells is likely due to an increased number of double negative memory B cells.

To dissect whether high levels of miR-155 in RA B cells are related to chronic inflammation and/or to B-cells maturation status, we analysed miR-155 expression in PB-derived B cells from autoantibody negative Psoriatic Arthritis (PsA) patients naïve to treatment with active systemic disease (DAS28 4.7±0.9) and compared to RA and HC. PsA patients have a lower proportion of memory CD19^+^ IgD^-^CD27^-^ cells (*P*=0.03, Mann-Whitney *U*-test) compared to RA (Supplementary Fig. 3). PB-derived B cells of PsA patients show miR-155 expression higher than HC (*P*=0.001, Mann-Whitney *U*-test) and comparable to ACPA-negative RA patients (*P*=0.25, Mann-Whitney *U*-test); but significantly lower than ACPA^+^ RA patients (Fig. 2f). These data suggest that expression of miR-155 in B cells can be associated with chronic inflammation; however, in RA patients is strongly related to ACPA positivity.

### miR-155 is abundantly expressed in B cells of RA synovium

**Figure 3:**
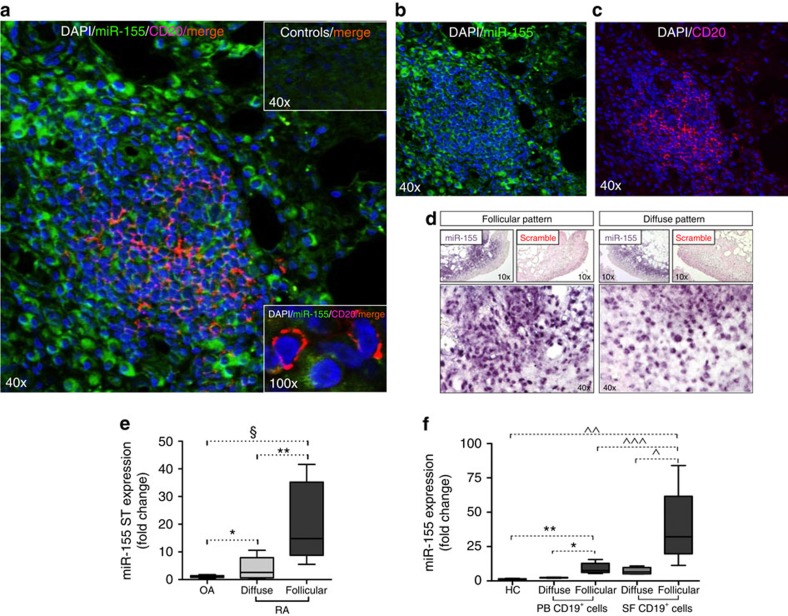
miR-155 expression in synovial RA B cells and in synovial tissue from RA and OA. (**a**–**c**) Double immunofluorescence staining of miR-155 (green) and CD20 (red) in RA synovial tissue with follicular histological pattern. (**a**) Representative picture for grade 3 lymphocyte aggregate^42^ (original magnification × 40; orange=double positive cells) with upper insert showing control staining (scramble probe/isotype) and a bottom insert showing × 100 magnification of miR-155^+^CD20^+^ cell; *n*=5. (**b**) Representative pictures of the distribution of miR-155 (green channel) and (**c**) CD20 (red channel) are shown. (**d**) Representative picture of miR-155 *in situ* hybridization on synovial tissue from RA with follicular (*n*=5) and diffuse (*n*=5) pattern (5-bromo-4-chloro-3′-indolyphosphate, purple); (**e**) qPCR assessing miR-155 expression in synovial tissue from RA with follicular (*n*=10) and diffuse (*n*=10) pattern and OA patients (*n*=5). *P=0.03 diffuse RA versus OA; **P=0.001 diffuse versus follicular RA; §*P*<0.0001 follicular RA versus OA patients. Data are shown as mean±s.d., 25–75 percentiles; Mann-Whitney *U*-test. (**f**) miR-155 expression in CD19^+^ cells isolated from PB and SF of RA patients with different synovial pattern; *P=0.01 PB CD19^+^ cells from follicular versus diffuse RA; **P=0.001 PB CD19^+^ cells from follicular RA versus HC; ^*P*=0.01 SF CD19^+^ cells from follicular versus diffuse RA; ^^*P*=0.001 SF CD19^+^ cells from follicular versus HC; ^^^*P*=0.02 SF CD19^+^ cells from follicular RA versus PB CD19^+^ cells from follicular RA; data are shown as mean±s.d., 25–75 percentiles; Mann-Whitney *U*-test. DAPI, 4′,6-diamidino-2-phenylindole.

To assess miR-155 expression in infiltrating B cells within the synovial tissue, we performed miR-155 fluorescent *in situ* hybridization on synovial tissues of RA patients. This demonstrated that synovial B cells abundantly express miR-155 (Fig. 3a-c). MiR-155 is preferentially overexpressed in synovial tissue of RA patients with follicular pattern, compared to those with diffuse pattern (Fig. 3d). This finding was confirmed on tissue lysates by qPCR. Synovial tissue of RA patients with a follicular pattern shows 3.96±4.19 fold higher expression of miR-155 compared to synovial tissue of RA patients with a diffuse pattern (*P*=0.001, Mann-Whitney *U*-test) and 20.54±14.52 fold more than OA (*P*<0.0001, Mann-Whitney *U*-test) (Fig. 3e). In addition, we found that RA patients with a synovial follicular pattern are characterized by CD19^+^ cells showing higher expression of miR-155 in PB and SF compartment compared to RA patients with diffuse pattern (*P*=0.03 and *P*=0.05 respectively, both calculated using Mann-Whitney *U*-test) (Fig. 3f). These observations suggest that miR-155 might be an important regulator of RA B-cell activation associated with autoantibody production.

### miR-155 is inducible in CD19^+^ cells

**Figure 4:**
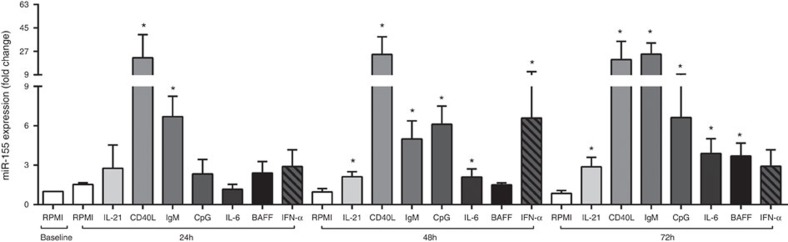
miR-155 in CD19^+^ cells is induced by inflammatory and maturation factors. miR-155 expression in PB CD19^+^ cells isolated from HC (*n*=5) after *in vitro* stimulation with CD40L (2 μg/ml), IL-21 (50 ng/ml), anti-IgM (10 μg/ml), CpG (1 μg/ml), Interferon alpha (400 IU/ml), IL-6 (30 ng/ml) and BAFF (20 ng/ml) for 24–72 h; *P<0.05. Data are shown as mean±s.d.; Mann-Whitney *U*-test. The data were generated in three independent experiments.

To dissect which mediators regulate miR-155 expression in human B cells, PB-derived CD19^+^ cells from healthy donors were stimulated with interleukin-21 (IL-21), CpG, CD40L, anti-immunoglobulin M (anti-IgM), IFN-α, IL-6 and B-cell activating factor (BAFF), which are known as activation and survival factors of B cells^10^,^11^,^12^,^13^,^14^,^15^,^16^ (*n*=5). We found that CD40L (2 μg/ml) and anti-IgM (10 μg/ml) are able to induce miR-155 expression in CD19^+^ cells already after 24 h of stimulation (22.03±30.91 and 6.70±2.69 fold increase *P*<0.05, respectively, Mann-Whitney *U*-test) and sustain the increase at later time points (48 and 72 h). While IL-21 (50 ng/ml), CpG (1 μg/ml) and IFN-α (400 IU/ml) induce miR-155 expression in CD19^+^ cells after 48 h of stimulation (2.12±0.85 fold with IL-21; 6.12±3.10 fold with CpG and 6.58±10.77 fold with IFN-alpha compared to baseline RPMI; *P*<0.05 respectively, all calculated using Mann-Whitney *U*-test) (Fig. 4). This increase persisted through 72 h. IL-6 and BAFF, which are regulators of B-cell activation^15^,^16^ and are present at high levels in SF compared to PB regardless of histological pattern in synovial tissues in RA patients (Supplementary Fig. 4) are also able to significantly increase miR-155 expression in CD19^+^ cells after 48 and 72 h stimulation (1.5±0.34 and 3.70±2.21 fold change after 48 and 72 h BAFF stimulation and 2.10±1.37; and 3.90±2.50 fold change after 48 and 72 h IL-6 stimulation) (Fig. 4). These data suggest that a wide range of inflammatory mediators and B-cell activators increase expression of miR-155 in B cells with the strongest impact mediated via maturation factors. Thus the cytokine and cell-rich RA synovial compartment is likely responsible for perpetuation of high levels of miR-155 in synovial B cells; and miR-155 may integrate at molecular levels distinct signals leading to B-cell activation.

### PU.1 is decreased in RA B cells within synovial compartment

**Figure 5:**
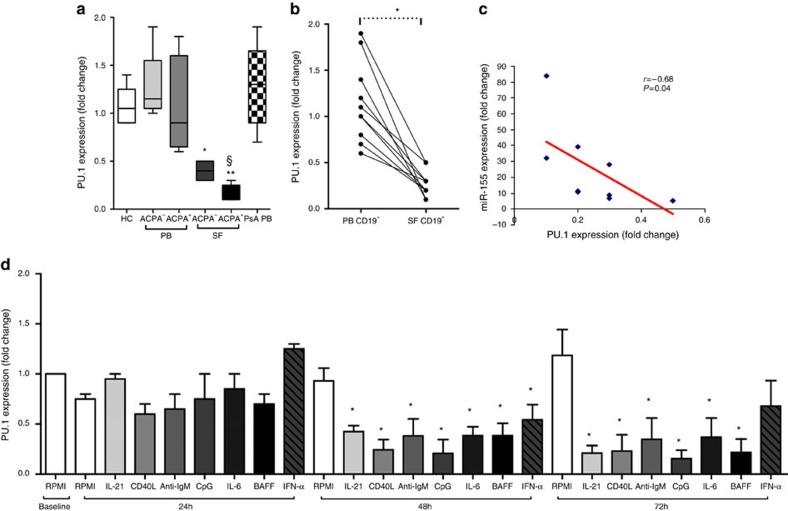
PU.1 expression in CD19^+^ cells of RA patients. (**a**) Expression of PU.1 in CD19^+^ cells isolated from PB and SF of RA patients compared to HC, **P=0.002 SF-derived CD19^+^ cells from ACPA^+^ RA versus HC; *P=0.001 SF-derived CD19^+^ cells from ACPA^-^ RA versus HC; and §*P*=0.001 SF-derived CD19^+^ cells from ACPA^+^ RA versus PB-derived CD19^+^ cells from ACPA^+^ RA; data are shown as mean±s.d., 25–75 percentiles; Mann-Whitney *U*-test. (**b**) Expression of PU.1 in CD19^+^ cells in paired RA PB and SF samples; *P=0.002; Wilcoxon test; (**c**) Inverse correlation between miR-155 and PU.1 expression in SF derived CD19^+^ cells in RA patients [*r*= −0.65; *P*=0.04; 95%CI (−0.38 to −0.88)]; Spearman Rank Correlation coefficient; (**d**) PU.1 expression in PB CD19^+^ cells isolated from HC (*n*=5) after *in vitro* stimulation with CD40L (2 μg/ml), IL-21 (50 ng/ml), anti-IgM (10 μg/ml), CpG (1 μg/ml), Interferon alpha (400 IU/ml), IL-6 (30 ng/ml) and BAFF (20 ng/ml) for 24–72 h; *P<0.05 CD40L, anti-IgM, IL-21, CpG, IFN-alpha, BAFF or IL-6 versus Baseline RPMI; data are shown as mean±s.d.; Mann-Whitney *U*-test.

Among miR-155 target genes, PU.1 was selected and evaluated in detail because it is crucial for early B-cell commitment and a negative regulator of B-cell maturation^9^,^17^,^18^,^19^. PU.1 is similarly expressed in PB B cells from healthy controls, RA patients (*P*=0.89, Mann-Whitney *U*-test) and PsA patients (*P*=0.81, Mann-Whitney *U*-test) (Fig. 5). Analysis of PU.1 expression in paired PB and SF samples of RA patients revealed that SF-derived CD19^+^ cells have lower PU.1 levels than PB-derived CD19^+^ cells (*P*=0.002, Wilcoxon test), consistent with a higher expression of its negative regulator miR-155 in the synovial compartment (Fig. 5b). Stratification of RA patients according to ACPA positivity revealed that SF-derived CD19^+^ cells of ACPA^+^ RA patients show significantly lower PU.1 expression compared to ACPA^-^ patients (Fig. 5a). An inverse correlation between miR-155 and PU.1 expression in SF-derived B cells [*r*= −0.65; *P*=0.04; 95%CI (−0.38 to −0.88) Spearman Rank Correlation coefficient] (Fig. 5c) further supports the presence of an active interaction between miR-155 and its target in RA B cells. Since negative regulation of PU.1 by miR-155 has been experimentally validated^20^, we next investigated PU.1 expression in B cell after stimulation with factors triggering miR-155 expression. We found that miR-155 is inducible by several factors including CD40L, anti-IgM, CpG, IL-21, IFN alpha, BAFF and IL-6. Conversely we found that PU.1 gene expression is significantly decreased after 48 and 72 h stimulation of CD19^+^ cells with CD40L, anti-IgM, CpG, IL-21, IL-6 and BAFF (Fig. 5d). In particular, PU.1 levels inversely correlate with miR-155 expression in healthy B cells after 72 h stimulation with CD40L [*r*= −0.99; *P*=0.0001; 95%CI (−0.98 to −1.00), Spearman Rank Correlation coefficient], CpG [*r*= −0.97; *P*=0.026; 95%CI (−0.85 to −1.00), Spearman Rank Correlation coefficient] and BAFF [*r*= −0.99; *P*=0.01; 95%CI (−0.66 to −1.00), Spearman Rank Correlation coefficient] respectively.

**Figure 6:**
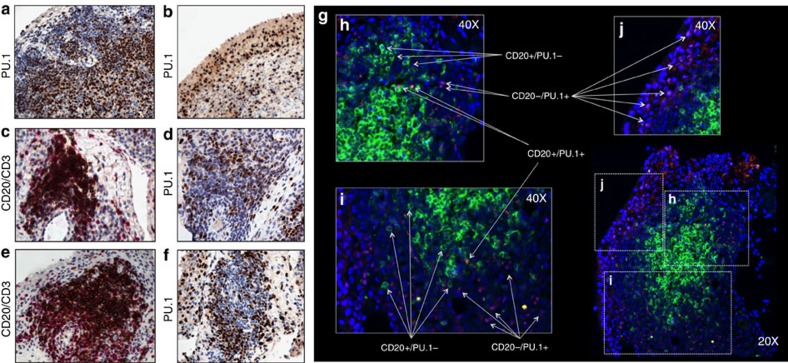
PU.1 expression in synovial tissue and synovial CD20^+^ cells of RA patients. (**a**–**d**) PU.1 Immunohistochemical staining (DAB) in synovial tissue of RA patients. (**a**) Representative PU.1 staining (Brown-DAB) in synovial tissue with diffuse pattern; (**b**) Representative PU.1 staining (Brown-DAB) in mononuclear cells in synovial lining of synovial tissue with diffuse pattern; (**c**,**e**) CD20 (DAB) and CD3 (RED) staining (original magnification × 20) and (**d**,**f**) PU.1 (DAB) staining in lymphocyte synovial follicules (original magnification × 20); (**g**) Representative immunofluorescence staining for PU.1 expression (TRITC, red) in synovial CD20^+^ cells (FITC, green) in RA patients (original magnification × 20); (**h**,**i**) PU.1 expression (TRITC) in CD20^+^ cells (FITC) within lymphocyte synovial aggregates (original magnification × 40); (**j**) PU.1 expression (TRITC) in the synovial lining layer (original magnification × 40). orange = double positive cells.

Immunohistochemical analysis demonstrated the presence of PU.1 in the synovial tissue with both a diffuse (Fig. 6a,b) and a follicular pattern (Fig. 6d,f). To quantitatively assess PU.1 expression in synovial tissue we performed RT-PCR on synovial tissue lysates, which revealed that RA patients with follicular synovitis have significantly less expression of PU.1 compared to RA patients with diffuse synovitis (*P*=0.02, Mann-Whitney *U*-test) (Supplementary Fig. 5). Moreover, we found significant inverse correlation between miR-155 and PU.1 expression in synovial tissue lysates in RA patients with follicular synovitis [*r*= −0.76; *P*=0.01; 95%CI (−0.58 – to −0.93), Spearman Rank Correlation coefficient]. To visualize PU.1 expression specifically in synovial B cells we performed double immunofluorescence for PU.1 and CD20 in synovial tissue biopsies of RA patients with follicular synovitis. Commensurate with qPCR data, only a few CD20^+^ cells within the ectopic lymphoid structures were positive for PU.1 (Fig. 6e).

All together these data support the notion that a miR-155-PU1 axis might be involved in RA B-cell activation.

### miR-155 inhibition in RA B cells reduces antibody synthesis

**Figure 7:**
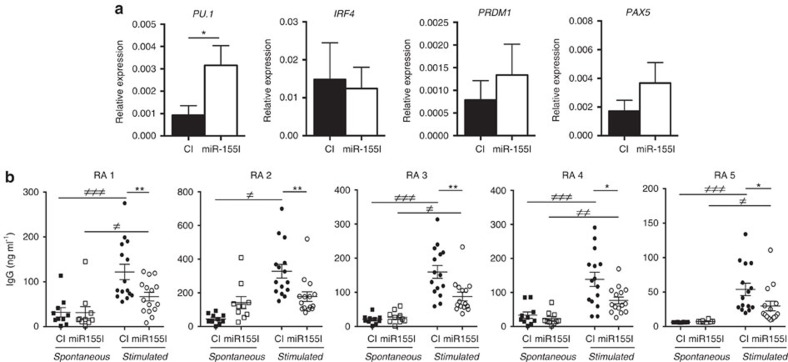
Inhibition of miR-155 in RA PB B cells reduces antibody production. (**a**) Inhibition of miR-155 in RA PB B cells derepresses PU.1. PB CD19^+^ cells from RA patients (*n*=6) were transfected with miR-155 inhibitor or control inhibitor (CI) using the Neon Transfection System. Expression of PU.1, IRF4, PRDM1, PAX5 and housekeeping 18S were determined by qPCR 48 h later. Data are presented as mean±s.e.m. of biological replicates. *P<0.05 anti-miR-155 versus CI; Wilcoxon test; (**b**) miR-155 inhibition reduces IgG production by RA B cells. RA PB CD19^+^ cells (*n*=5) were transfected with miR-155 inhibitor or control mimic and stimulated with plate bound human CD40 ligand (2 μg/ml) in the presence of BAFF (100 ng/ml), IL-21 (50 ng/ml) and anti-IgM F(ab)2-fragments (5 μg/ml) or left in the presence of BAFF and IL-6 (spontaneous production of antibodies). Each condition was performed in *n*=10 (spontaneous) and *n*=14 replicates (stimulated). * *P*<0.05 stimulated miR-155 inhibitor transfected cells versus stimulated control transfected cells;≠*P*<0.05 stimulated versus spontaneous production of antibodies; Wilcoxon test. The data was generated in three independent experiments.

To functionally evaluate the role of miR-155 in RA B-cell activation, the expression of its target genes and production of IgG were evaluated in RA PB B cells transfected with a control or miR-155 inhibitor. Patient clinical data and B-cell transfection efficiency, which was above 60%, are shown in Supplementary Table 1a-b and Supplementary Fig. 6. RA B cells (*n*=6) transfected with anti-miR-155 show a significant increase of PU.1 expression (*P*<0.05, Wilcoxon test) compared to RA B cells treated with control inhibitor at 48 h time point (Fig. 7). There were no significant changes in the expression of other B-cell-specific transcription factors (IRF4, PRDM1 and PAX5), which were previously reported to be under direct or indirect control of miR-155^9^,^21^,^22^ (Fig. 7a). These data suggest that PU.1 is regulated by miR-155 in RA B cells and that inhibition of miR-155 could impact the antibody production. To test the latter possibility, PB CD19^+^ cells from ACPA^+^ RA patients (*n*=5) were transfected with miR-155 or control inhibitor and stimulated with anti-IgM, CD40L, IL-21 and BAFF or left unstimulated for 7 days. Stimulation strongly increases total IgG production by RA B cells (Fig. 7b) and miR-155 inhibition leads to a substantial decrease in antibody production compared to control inhibitor treated cells (Fig. 7b). These data suggest that miR-155 in RA B cells is a key post-transcriptional regulator of antibody production.

## Discussion

RA is an autoimmune disease that affects nearly 1% of the population and can lead to loss of function, disability and increased mortality. To date, long lasting, usually drug-maintained disease remission can be achieved in about 40–50% of cases. Thus there is considerable unmet need to fully understand the disease process in order to improve either use of available therapeutics or elaboration of novel targets. In RA, immunological dysregulation exemplified by, for example, ACPA serological positivity and serum cytokine/chemokine profiles, reflects loss of immunological tolerance to self-antigen, and can be detected several years before the clinical onset of arthritis^23^. This places B cells in the centre of pathogenesis of RA. B-cell depletion therapy is effective in a proportion of RA patients^24^. A critical stage of effector B-cell development is defined by GC formation. During the GC reaction, B cells undergo somatic hypermutation of their immunoglobulin variable regions and class-switch recombination. B cells that have acquired the ability to express high affinity immunoglobulins are then positively selected and further differentiate into final effectors of the humoural immune response, that is, memory B cells and plasma cells contributing to immune response by antibody release, antigen presentation and by releasing a wide range of cytokine and chemokines^25^.

Synovial infiltration by inflammatory cells occurs with different degrees in RA: from diffuse to simple cellular aggregates, to real pseudo-follicular structures resembling GC (lymphoid neogenesis). Despite active research there are still controversial data on this issue^3^,^26^. Because of synovial infiltrates variability, it is mandatory to distinguish between lymphoid aggregation and lymphoid neogenesis^27^. It has been proposed that synovial tissue with ectopic lymphoid neogenesis characterizes a distinct physiopathologic subtype of RA patients and that its presence is related to synovial inflammation in early RA patients and can change over time during the disease course^28^. Our study suggests that synovial follicular structures are present during an early disease phase and can be detected with a similar frequency in RA patients responding and not responding to methotrexate treatment, consistent with prior studies^27^. The biological activity of the synovial aggregate structures is still undefined—contradictory data on their role in the promotion of autoimmunity, chronic inflammation and driving the production of pathogenetic autoantibodies exist: Humby *et al* demonstrated that synovial follicular units in RA express activation-induced cytidine deaminase and are surrounded by ACPA-producing plasma-cells^3^, whereas Cantaert *et al* demonstrated that ectopic lymphoid neogenesis is not directly associated with the local production of ACPA and RF in RA joints^29^. Our findings support those of Humby *et al* since we found a significantly higher incidence of synovial aggregate pattern and significantly higher percentages of resident CD20^+^, CD68^+^ and CD3^+^ cells in ACPA-positive patients compared to ACPA-negative ones.

Several lines of evidence suggest an important role for specific miRNAs in B-cell function^8^,^20^,^30^,^31^,^32^,^33^. MiRNA deregulation was found in several B-cells-mediated diseases. In mouse models, miR-155 has been demonstrated to affect regulation of the GC response through modulation of cytokine production^4^ and optimal antibody response^8^,^20^,^30^,^31^,^32^,^33^. Moreover, miR-155 overexpression is associated with aggressive forms of diffuse large B-cell lymphomas^34^. We have previously shown that miR-155 has a crucial role in the development of experimental arthritis^6^. MiR-155^−/−^ mice are resistant to collagen-induced arthritis associated with lower levels of anti-collagen antibodies suggesting a central role of miR-155 in loss of tolerance and in promoting B-cell-driven autoimmune processes^6^. Herein, we provide for the first time evidence of the overexpression of miR-155 in B cells of RA patients in the PB and in the synovial compartment. In particular, we found that the expression of miR-155 in RA B cells is already increased and peaks in early phases of the disease; mainly in double negative (IgD^-^/CD27^-^) memory B cells. This population was described previously to be increased in autoimmune diseases, including SLE and RA^35^,^36^ and to be important for IgG^+^ plasmablast generation^37^. The association between miR-155 expression in B cells and auto-antibody production is suggested by the significant higher expression of miR-155 in B cells isolated from ACPA-positive RA patients compared to ACPA-negative ones, particularly in the early phases of the disease and in patients with follicular synovitis. These data are commensurate with *in vitro* data showing that maturation of B cells towards antibody-producing cells (anti-IgM, CD40L stimulation) had the most significant impact on miR-155 expression in B cells. Inflammatory mediators, including IL-6, BAFF and IL-21 also increased miR-155 expression suggesting that the overall inflammatory milieu contributes to miR-155 expression in B cells. This was supported by the presence of elevated levels of miR-155 in B cells of PsA patients with high disease activity. However, these levels were comparable to those observed in ACPA-negative RA patients, implicating that maturation of B cells towards antibody-producing cells is the key factor in miR-155 expression in RA.

RA synovium likely comprises a niche in which expanded autoreactive B-cells and autoreactive plasma cells can reside^38^. B-cell memory is supported by plasma cells, responsible for antibodies production, and memory B cells, representing precursors able to replace plasma cells through antigen-dependent and independent mechanisms^39^. This maturation cascade of B cells is regulated by miR-155 in physiologic condition with a progressive upregulation of miR-155 from naïve to memory committed B cells^4^. We found that most of the resident synovial CD20^+^ cells in synovial follicules were miR-155 positive. Among the possible molecular mechanisms by which miR-155 can influence B-cell activation, we investigated the ets-family transcriptional regulator PU.1. Vigorito *et al.* provided strong evidence that mRNA encoding PU.1 is a miR-155 target^20^. PU.1 mRNA is elevated in miR-155-deficient mouse B cells and miR-155 directly targets PU.1 mRNA via a predicted miR-155-binding site in the 3′ untranslated region. Moreover, they showed that forced expression of PU.1 in cultured wild-type mouse primary B cells recapitulates the miR-155-deficient defect in IgG1 production. It is known that PU.1 is important for early B-cell commitment and is downregulated during the maturation of B cells^17^,^18^,^19^. The levels of PU.1 are high in GC B cells but are reduced upon plasmacytic differentiation^40^. In our study we assessed for the first time the expression of PU.1 in B cells derived from multiple biological compartments in RA patients finding that SF-derived CD19^+^ cells from RA patients show lower expression of PU.1 compared to healthy controls, consistently with miR-155 overexpression. Lu *et al* demonstrated that PU.1 is a negative regulator of Ig secretion in animal models^9^. In line with this, our study revealed that SF-derived B cells of RA patients show lower expression of PU.1 in ACPA-positive compared to ACPA-negative RA patients and a low number of PU.1 positive cells among resident CD20^+^ cells within synovial follicular structures in RA patients. The functional analysis of miR-155 in RA B cells revealed that inhibition of endogenous miR-155 in these cells led to the restoration of PU.1 expression and inhibited B-cell receptor (BCR) crosslinking induced antibody production.

**Figure 8:**
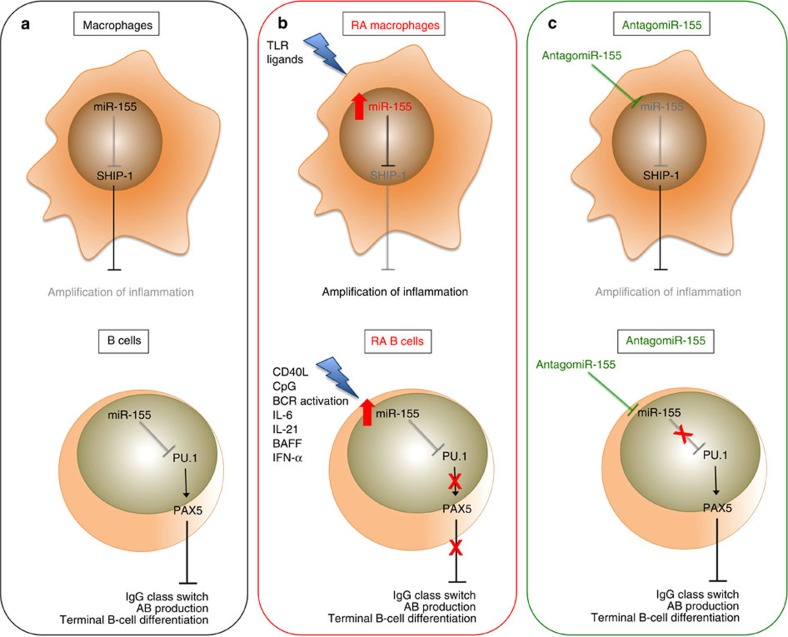
Rationale for the use of antagomiR-155 in the treatment of RA synovitis. (**a**) miR-155 control of SHIP-1 and PU.1 is crucial for the regulation of inflammatory response in macrophages and for antibody production and terminal differentiation of B cells; (**b**) In RA synovial compartment, macrophages and B cells are characterized by higher expression of miR-155 due to endogenous TLR ligands^6^ and other soluble factors, for example, CD40L, IL21, CpG, IFN-alpha, IL-6 and BAFF. This leads to the downregulation of SHIP-1 and PU.1 expression and thus to the amplification of the inflammatory cascade by macrophages and increase in antibody production and B-cell maturation; (**c**) Thus, the administration of antagomiR-155 could restore the normal expression of SHIP-1 and PU.1 acting both on macrophages and B cells and facilitate the resolution of autoimmunity and inflammation in RA. SHIP-1, inositol polyphosphate-5-phosphatase; PU.1, Spi-1 proto-oncogene; TLR, Toll like receptor BAFF, B-cell activating factor; CD40L, CD40 ligand, BCR, B-cell receptor.

In conclusion, our study suggests that RA B-cell antibody production is controlled by the miR-155-PU1 pathway. Since miR-155 has been previously demonstrated to be a regulator of myeloid cell activity^6^ and of T cells^41^, we propose that an inhibitor of miR-155 could be used to simultaneously treat both myeloid and lymphoid synovial phenotypes^1^, increasing the current response rate of RA patients (Fig. 8a-c). This is predicated however on finding sufficiently safe modalities for miR-155 inhibition.

## Materials and methods

### Patient characteristics

Sixty consecutive patients fulfilling the American College of Rheumatology 2010 revised criteria for RA^42^ were enrolled in the study at the Division of Rheumatology, Fondazione Policlinico Universitario A. Gemelli, Catholic University of the Sacred Heart in Rome. As comparison groups we included in the study patients (*n*=14) with osteoarthritis (OA) and healthy control subjects (*n*=9) [7(78%) female; 54.7±8.6 years-old] (HC). RA patients were divided into early (ERA) (*n*=27) and long standing (LSRA) (*n*=33) based on the disease duration (more or less than 1 year). All ERA patients were naïve to any pharmacological treatment whereas all LSRA were taking stable dose of Methotrexate (mean dose: 12.9±6.1 mg/week). PsA patients^43^ (*n*=5) with active disease (DAS28 4.7±0.9) due to peripheral joints arthritis without spine involvement, and naïve to treatment were enrolled as a comparison group. Clinical and laboratory evaluations included the Disease Activity Score (DAS28), the number of tender and swollen joints at 28 sites, the ESR and C-reactive protein. All PB samples were tested for IgM and IgA Rheumatoid Factor (RF) isotypes (Orgentec Diagnostika, Dundee, UK) and ACPA antibodies (Diastat, Axis-Shield Diagnostics, Ltd., Dundee, UK). The study protocol was approved by the local Ethic Committee of the Catholic University of the Sacred Heart and by the West of Scotland Research Ethical Committee (11/S0704/7). All subjects provided signed informed consent.

### Immunohistochemistry on synovial tissues

All enrolled patients underwent ultrasound guided synovial tissue biopsy of the knee. All patients underwent ultrasound evaluation of the knee using an ultrasound machine with a multifrequency linear transducer (MyLab Twice, Esaote). Using the ultrasound view, the best point of entrance for the needle was identified on the lateral margin of the suprapatellar recess. Each patient was provided with a face-mask and cap and the whole procedure was undertaken under sterile conditions. Skin disinfection was done with iodine solution (performed twice, starting from the point of needle entrance up to 25 cm proximally and distally). Arthrocentesis of the knee joint was performed using the lateral suprapatellar access if joint effusion was present. The skin, subcutaneous tissue and joint capsule was anaesthetised with 10 ml lidocaine 2%. Next, a 14G needle (Precisa 1410-HS Hospital Service Spa, Italy) was inserted into the joint. Regions of synovial hypertrophy were identified under grey-scale guidance^44^ to ensure sampling of representative synovial tissue. All the synovial tissue specimens obtained (at least eight pieces for each analysis) were placed on a nonwoven wet gauze for collection^44^. Once collected, synovial tissue specimens were fixed in 10% neutral-buffered formalin and embedded in paraffin for histology or stored in Qiazol for RNA isolation. Briefly, paraffin-embedded ST specimens were sectioned at 3–4 μm. Firstly, sections were stained for Hematoxylin and Eosin as follows: sections were deparaffinized in xylene and rehydrated in a graded ethanol series. Then, they were stained in hematoxylin and counterstained in Eosin/Phloxine. Finally, sections were dehydrated, cleared in xylene and mounted with Bio Mount (Bio-Optica). Other sections were stained with IgG2a mouse anti-human monoclonal antibody for CD68 (clone 514H12; antibody at 6.7 mg/ml) or IgG1 mouse anti-human monoclonal antibody for CD20 (clone L26; at 1.2 mg/ml) or IgG1 mouse anti-human monoclonal antibody for CD3 (clone LN 10; at 1.0 mg/ml),or IgG2a mouse anti-human monoclonal antibody for CD21 (clone 2G9; at 0.34 mg/ml; all from Leica Biosystem, Newcastle, UK), or IgG rabbit monoclonal anti-human for PU.1 (#2266; dilution 1/100, Cell Signalling Technology) by immunostainer BOND MAX III (Leica, Newcastle, UK).

Double immunohistochemical staining for CD21/CD68 and CD20/CD3, or single immunohistochemical staining for PU.1 was performed as follows: 3-μm-thick sections were prepared from formalin-fixed paraffin-embedded tissue blocks and were dried in a 60 °C oven for 30 min. The sections were placed in a Bond Max Automated Immunohistochemistry Vision Biosystem (Leica Microsystems GmbH, Wetzlar, Germany) according to the following protocol: firstly, tissues were deparaffinized and pre-treated with the Epitope Retrieval Solution 1 (CITRATE buffer) or Solution 2 (EDTA-buffer) at 98 °C for 10 min according to the manufacturer's instructions. After washing, peroxidase blocking was carried out for 10 min using the Bond Polymer Refine Detection Kit DC9800 (Leica Microsystems GmbH). Tissues were again washed and then incubated with the primary antibody for 30 min. Subsequently, tissues were incubated with polymer for 10 min and developed with DAB-Chromogen or RED-Chromogen and finally counterstained with hematoxylin^45^.

Slides were examined using a light microscope (Leica DM 2000). RA and OA tissues were classified as diffuse or follicular based on the immunostaining of CD68, CD21, CD20 and CD3 positive cells^46^. RA and OA tissues were evaluated using a numerical score based on the number of positive cells in the lining and sublining areas of the section (three different fields in each section), with a score of 0 indicating no positive cells; 1 indicating <10% positive cells; 2 indicating 10–50% positive cells; and 3 indicating >50% positive cells^6^. —Six to eight samples were collected and evaluated for each patient, to reduce the sampling error^44^.

### *In situ* hybridization on synovial tissues

After deparaffinization, sections were treated with proteinase-K (15 μg/ml) at 37 °C for 10 min. Hybridization with 5 nmol Locked Nucleic Acid 5′ and 3′ digoxigenin (DIG)-labelled control scramble (GTGTAACACGTCTATACGCCCA) or miR-155–specific (TATCACGATTAGCATTAA) probes (both from Exiqon) was performed at 50 °C for 1 h. After hybridization, sections were washed in 5 × SSC at 50 °C for 5 min, in 1 × SSC at 50 °C for two washings of 5 min each, in 0.2 × SSC at 50 °C for two washings of 5 min each, and in 0.2 × SSC at RT for 5 min. Next, sections were blocked with DIG Wash and Blocking buffer (Roche) in a humidifying chamber at room temperature (RT) for 15 min. Slides then were incubated with alkaline phosphatase-conjugated sheep anti-DIG antibody (1:800; Roche) in blocking solution supplemented with 2% sheep serum for 1 h at RT. Sections were incubated with nitro-blue tetrazolium and 5-bromo-4-chloro-3′-indolyphosphate solution (Roche) at 30 °C for 2 h. To stop the reaction, slides were washed twice for 5 min with buffer containing 50 mM Tris-HCl, 150 mM NaCl and 10 mM KCl. Nuclear counterstaining was performed using Nuclear Fast Red (Vector).

Some sections were incubated with fluorescein-conjugated anti-DIG antibody (Roche) followed by incubation with monoclonal mouse anti-human CD20 antibody (at 1 μg/ml; DakoCytomation) at 4 °C overnight. On the next day, sections were incubated with biotinylated horse anti-mouse IgG (H+L; Vector) for 30 min at RT followed by incubation with Texas Red Avidin D (Vector) at RT for 30 min. Negative control slides were prepared in the same manner using mouse nonspecific IgG1 (DakoCytomation). The sections were analysed using a fluorescent imaging microscope (LSM 510 Meta confocal microscope (Zeiss, Cambridge, UK)) and the images captured using Zeiss imaging acquisition software. Signal from controls was subtracted from the signal of miR-155/CD20 staining.

### Fluorescent immunohistochemistry on synovial tissue

Double immunofluorescence staining was performed on formalin-fixed RA synovial tissues with a primary antibody against CD20 (clone L26 mouse anti-human monoclonal antibody, at 1.2 mg/ml, Leica Biosystem, Newcastle, UK) and anti-PU.1 (rabbit monoclonal anti-human PU.1, #2266; dilution 1/100, Cell Signalling Technology) at 37 °C for 1 h. Sections then were rinsed and incubated with the appropriate secondary conjugated antibodies (Fluorescein isothiocyanate (FITC) conjugated goat anti-mouse IgG H&L, #ab6785, (Abcam) (dilution 1/1000) and Tetramethylrhodamine isothiocyanante (TRITC) conjugated goat anti-rabbit IgG H&L, #ab6718, Abcam (dilution 1/1000)) at RT for 1 h. Antigen retrieval was performed by microwaving in a citric acid-pH 9.2. To minimize nonspecific antibody binding, slides were pre-incubated with phosphate-buffered saline 10% bovine serum albumin (BSA) for 30 min. Slides were mounted in medium containing DAPI (H-1200; Vector) and were scanned on an APERIO fluorescent microscope (Leica Biosystems).

### FACS of B cells in PB and SF

Fresh PB or SF samples were processed within a few hours after sample collection. One hundred microlitres of whole PB in EDTA, or 5 × 10^5^ mononuclear cells derived from SF (separated by Ficoll-Hipaque (Cederlane, Ontario, Canada) density gradient centrifugation) in 100 μl of phosphate-buffered saline were first incubated in the dark at RT for 20 min with anti-human antibodies specific for CD45 (APC-A750) (clone J33) (dilution 1/25), CD19 (APC-700) (clone J3-119) (dilution 1/25), CD38 (PC5 or APC) (clone LS198-4-3) (dilution 1/25), CD27 (PC7) (clone 1A4CD27) (dilution 1/25) and FITC-conjugated IgD (clone IA6-2) (dilution 1/25) (all by Beckman Coulter, Marseille France). After staining, the cells were fixed, washed and erythrocytes were lysed. Samples were immediately analysed on optimally compensated 8 color Navios flow-cytometer and data were analysed with Kaluza software (Beckman Coulter, Marseille, France). Lymphocytes were gated on the basis of forward and side-scatter light properties (confirmed by CD45 staining) and at least 10,000 CD19^+^ cells were analysed. B-cell subsets were evaluated by the expression of surface B-cell markers according to IgD/CD27 classification^47^.

### miR-155 expression in CD19^+^ cells by qPCR

Peripheral blood (HC, RA, PsA) and synovial fluid mononuclear cells (PBMCs/SFMCs) were isolated by density gradient centrifugation on histopaque. CD19^+^ cells were then isolated from the mononuclear preparation using CD19 MACS MicroBeads (Miltenyi Biotech), and total RNA from the purified CD19^+^ cells was isolated using the miRNeasy kit (Qiagen). The CD19^+^ cells purity was assessed by FACS analysis (mean±s.d.; 97.0±0.7). The miScript Reverse Transcription Kit (Qiagen) was used for cDNA preparation. Expression of miR-155 and U1/U6 (housekeeping controls) was evaluated using the SYBR Green method using human miR-155 (MS00003605) and U1 (MS00013986) or U6 (MS00033740) specific primers (Qiagen).

### miR-155 expression in RA B-cell subsets sorted from PB

CD19^+^ B cells were enriched from PBMCs from RA patients (*n*=6) (Supplementary Table 1c) using CD19 microbeads and Auto-MACS separator according to the manufacturer's protocol (Miltenyi Biotec). Purified CD19^+^ cells were then stained with a viability marker (Fixable Viability Dye eFluor^®^ 780, eBioscience) for 20 min followed by staining with fluorochrome-labelled antibodies: FITC anti-human CD27 (clone M-T27) (#356404, dilution 1/20, Biolegend), PE/Cy7 anti-human CD38 (clone HB-7) (#356608, dilution 1/20, Biolegend), APC/H7 mouse anti-human IgD (clone IA6-2) (#561305, dilution 1/20, BD Bioscience) and PerCP-Cy5.5 mouse anti-human IgM (clone G20-127) (#561285, dilution 1/20, BD Bioscience). Using recommended isotype controls the specificity of the staining was assured. Cells were incubated with antibodies or isotypes for 30 min at 4 °C. Purified B-cell subsets were sorted using FACS Aria III using a 75 mm nozzle and the expression of miR-155 was evaluated as described above.

### PU.1 expression in CD19^+^ cells from RA patients by qPCR

CD19^+^ cells were isolated from paired PB and SF samples from 10 RA patients. An iScript^TM^ cDNA Synthesis Kit (Bio Rad Laboratories, Hercules, CA) was used for cDNA preparation. A FastStart Universal Probe Master (Roche Diagnostics, Germany) was used for qPCR using the following primers/probes: human PU.1 (100053625) and Glyceraldehyde 3-phosphate dehydrogenase (GAPDH, 100015432) both from Roche Diagnostics, Germany.

### Plasma/synovial fluid IL-6 and BAFF

IL-6 and BAFF in plasma and SF (if available) of RA subjects were measured by ELISA kit following the instructions of the manufacturer (R&D Systems, UK). The sensitivity of the test was 0.70 pg/ml for IL-6 and 2.68 pg/ml for BAFF respectively.

### miR-155 and PU.1 expression in stimulated CD19^+^ cells

CD19^+^ cells were purified from PBMCs of five healthy control subjects [49.2±12.1 years old, 4(80%) female] using CD19 MACS MicroBeads (Miltenyi Biotech). CD19^+^ cells were suspended at 5 × 10^5^ in 0.5 ml of complete culture medium (RPMI with 10% FCS, 100 U/ml penicillin and 100 μg/ml streptomycin) and were incubated in the wells of a 48-well culture plate with or without CD40L (2 μg/ml; Enzo Life Sciences), IL-21 (50 ng/ml; R&D), anti-IgM (10 μg/ml; Jackson ImmunoResearch), CpG (1 μg/ml; InvivoGen), IFN-alpha (400 IU/ml; pbl assay science), IL-6 (30 ng/ml; R&D) or BAFF (20 ng/ml; R&D) for 24, 48 or 72 h at 37 °C in 5% CO_2_. After stimulation, CD19^+^ cells were collected and the RNA was harvested, and the miR-155 and PU.1 expression was assessed by qPCR as described above

### Transcription factors expression in RA B cells

Purified CD19^+^ cells from RA patients (*n*=6; Supplementary Table 1a) were transfected with either hsa-miR-155-5p inhibitor (miR-155I) or control inhibitor (CI) using the Neon Transfection System with protocol number 2 (Voltage=2000, Pulse number=2). Transfected cells were seeded in 24-well plates at a concentration of 2 × 10^6^ cells in 1 ml of complete medium (RPMI 1640 medium; Invitrogen) supplemented with 10% FCS, 100 U/ml penicillin and 100 μg/ml streptomycin L-Glutamine (2 mM) in the presence of IL-6 (30 ng/ml) and BAFF (20 ng/ml). Cells were incubated at 37 °C in 5% CO_2_ for 24 and 48 h respectively. Cell viability and transfection efficiency were evaluated with Fixable Viability Dye eFluor 780 and Dy547 labelled control inhibitor respectively (Supplementary Fig. 6a,b). The total RNA was reverse transcribed into cDNA using the High Capacity cDNA reverse transcription kit (Applied Biosystems, UK). Quantitative PCR was performed on triplicate samples using the ABI 7900HT Fast Real Time PCR System (Applied Biosystems, UK) with TaQman probe and primers; HS00277134_m1 PAX5, HS00153357_m1PRDM1, HS02786711_m1SPI1 and SYBR Green primers: IRF4 FW 5′- ACC GGC AGA TGT CCA TGA G- 3′ and IRF4 Rev: 5′-GTG GCA TCA TGT AGT TGT GAA CCT -3′. The expression of target genes was normalized to the endogenous control 18S.

### IgG production by CD19^+^ cells upon miR-155 manipulation

PB CD19^+^ cells from ACPA-positive RA patients (*n*=5, Supplementary Table 1B; Supplementary Fig. 6a,b) were transfected with miR-155 inhibitor or control inhibitor as described above and cultured at a density of 2 × 10^4^ cells/well in IMDM supplemented with 10% FCS, penicillin/streptomycin (100 U/ml) and 2 mM Glutamax in 96-wells flat bottom plates. Transfected cell were either stimulated with plate bound human CD40 ligand (2 μg/ml) and anti-IgM (5 μg/ml) in the presence of BAFF (100 ng/ml), IL21 (50 ng/ml) or left unstimulated in media supplemented in survival factors: IL-6 (30 ng/ml) and BAFF (20 ng/ml) for 7 days as described by Kerkman *et al*^48^. Each condition was performed in 14-10 replicates. Standard ELISA was used to assess the level of total IgG in culture supernatants.

### Statistical analysis

Statistical analysis was performed using SPSS 20.0 (SPSS, Chicago, IL) and Prism software (Graph-Pad, San Diego, CA). Categorical and quantitative variables were described as frequencies, percentage and mean±standard deviation (s.d.). Data on demographic and clinical features were compared between patients using the non-parametric Mann-Whitney U test or χ^2^ test, as appropriate. The Wilcoxon test was used to compare cytokines levels between matched PB and SF. For the semi-quantitative determination of the expression of human miR-155 and the control U1 in CD19^+^ cells, ΔCt values were generated after subtraction from the gene of interest (miR-155) Ct value of controls (U1). The ΔΔCt values were obtained by normalizing the ΔCts with the mean ΔCt of healthy control group. For the semi-quantitative determination of the expression of human PU.1 and the control GAPDH, ΔCt values were generated, after subtraction from the gene of interest (PU.1) Ct value of controls (GAPDH). The ΔΔCt values were obtained by normalizing the ΔCts with the mean ΔCt of healthy control group or the mean value of RA patients with diffuse synovitis. Relative quantitative values for the expression of miR-155 in B cell subsets and transcription factors in cultured B cells were calculated as 2̂−ΔCt, after subtraction from the gene of interest (miR-155/transcription factors) Ct value of controls (U6 or 18S). Spearman's rank correlation test was used for correlation in all analyses. A *P* value <0.05 was considered statistically significant.

### Data availability

The data that support the findings of the study are available from the corresponding author upon request.

## Additional information

**How to cite this article:** Alivernini S, *et al.* MicroRNA-155 influences B-cell function through PU.1 in rheumatoid arthritis. *Nat. Commun.* **7,** 12970 doi: 10.1038/ncomms12970 (2016).

## Supplementary Material

Supplementary Information Supplementary Figures 1-6 and Supplementary Table 1

## Figures and Tables

**Table 1:** Demographical, clinical and immunological parameters of enrolled RA, PsA and OA patients (tissue phenotyping, miR-155 expression in CD19^+^ and in tissues).

Parameters	OA cohort (*n*=14)	PsA cohort (*n*=5)	RA cohort (*n*=60)	ERA (*n*=27)	LSRA (*n*=33)	*p*
Female, *n* (%)	10 (71.4)	3 (60.0)	47 (78.3)	20 (74.1)	27 (81.8)	*0.47*
Age, years (mean±s.d.)	64.0±7.3	56.8±11.4	55.9±13.9	51.2±13.2	59.5±13.6	*0.61*
Disease duration, months (mean±s.d.)	4.8±2.6	7.3±3.1	11.7±4.8	6.7±5.6	15.6±3.9	***0.001***
DAS28 (mean±s.d.)	−	4.7±0.9	4.9±1.2	5.0±1.3	4.81±1.12	*0.89*
ACPA positivity, n (%)	0 (0.0)	0 (0.0)	32 (53.3)	15 (55.6)	17 (51.5)	*0.76*
Follicular synovial pattern, *n* (%)	0 (0.0)	2 (40.0)	29 (48.3)	14 (51.8)	15 (45.5)	*0.62*
ESR, mm/1st hour(mean±s.d.)	26.1±20.6	42.7±23.8	45.5±32.8	50.3±35.7	41.5±30.1	*0.06*
CRP, mg/dL(mean±s.d.)	9.0±11.0	19.8±21.6	20.3±23.5	19.6±18.0	20.9±27.6	*0.79*

ACPA, anti-citrullinated peptides antibodies; CRP, C-reactive protein; DAS, disease activity score; ERA, early RA; ESR, erythrocyte sedimentation rate; LSRA, long-standing RA; OA, osteoarthritis; PsA, psoriatic arthritis; RA, rheumatoid arthritis.*P*=*ERA* versus *LSRA patients.* Bold: statistically significant. *P* values were evaluated by Mann-Whitney *U*-test or *χ*^2^ test as appropriate.
