# Supplementary material for: MicroRNA-155 influences B-cell function through PU.1 in rheumatoid arthritis
Source: Nat Commun. 2016 Sep 27;7:12970. doi: 10.1038/ncomms12970 (PMC5052655; doi:10.1038/ncomms12970)
Supplement: Supplementary Information — Supplementary Figures 1-6 and Supplementary Table 1 [file ncomms12970-s1.pdf]

Supplementary Figure 1

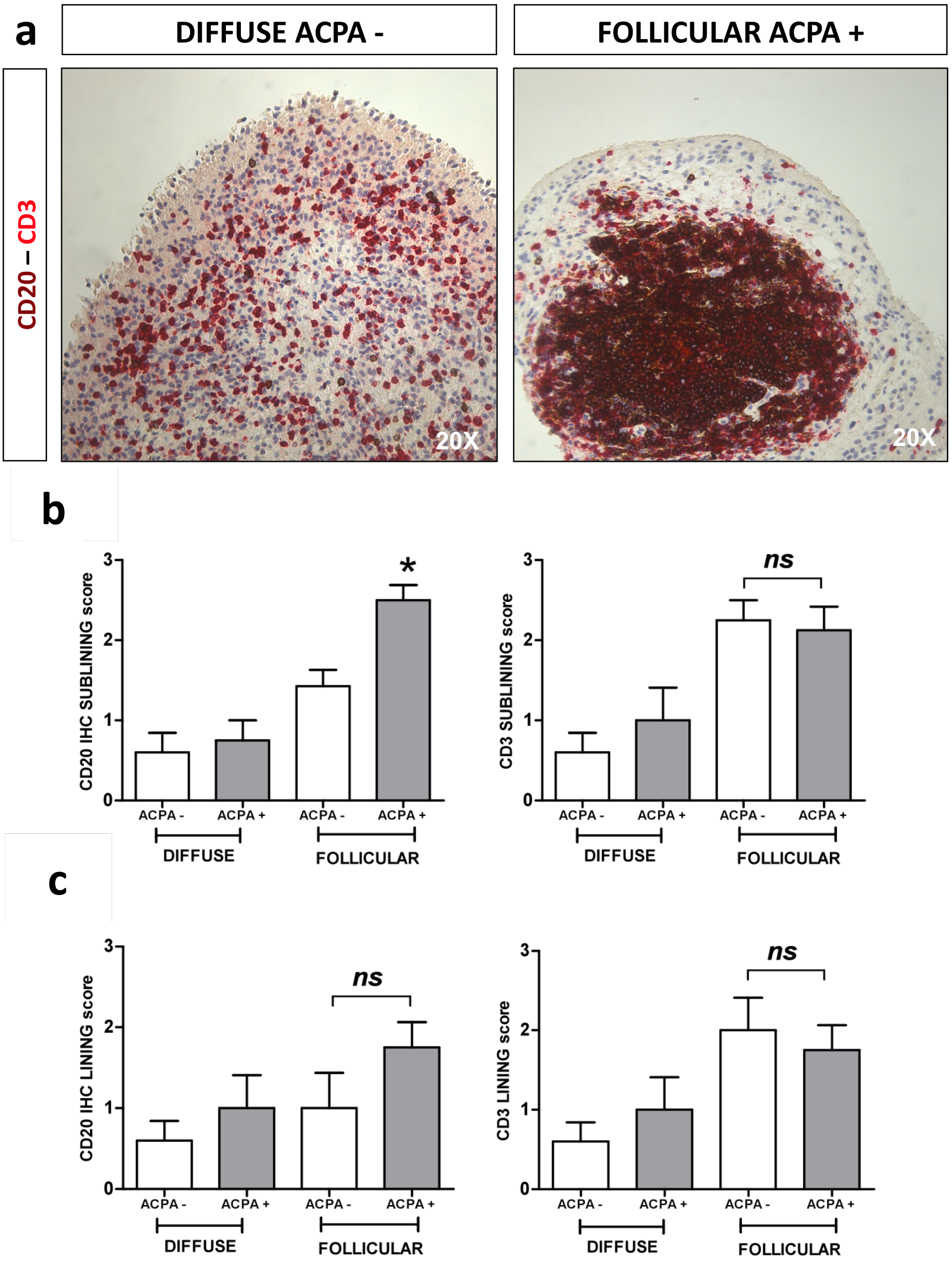

Supplementary Figure 1a,c. CD20 and CD3 double immunohistochemistry in RA synovial tissue with ACPA negative diffuse and ACPA positive follicular patterns; **(a)** Representative staining of CD20 (Brown-DAB) and CD3 (RED) in RA synovial tissue with ACPA negative diffuse and ACPA positive follicular patterns (Magnification 20X); **(b)** CD20 staining score for lining and sub-lining areas in RA synovial tissue with diffuse (n=15) and follicular (n=15) synovial pattern; *p=0.01 Diffuse ACPA$^-$ vs Follicular ACPA$^+$; Data are mean ± SD; Mann-Whitney U-test; **(c)** CD3 staining score for lining and sub-lining areas in RA synovial tissue with diffuse and follicular synovial pattern; Data are mean ± SD, ns: not significant, Mann-Whitney U-test.

Supplementary Figure 2

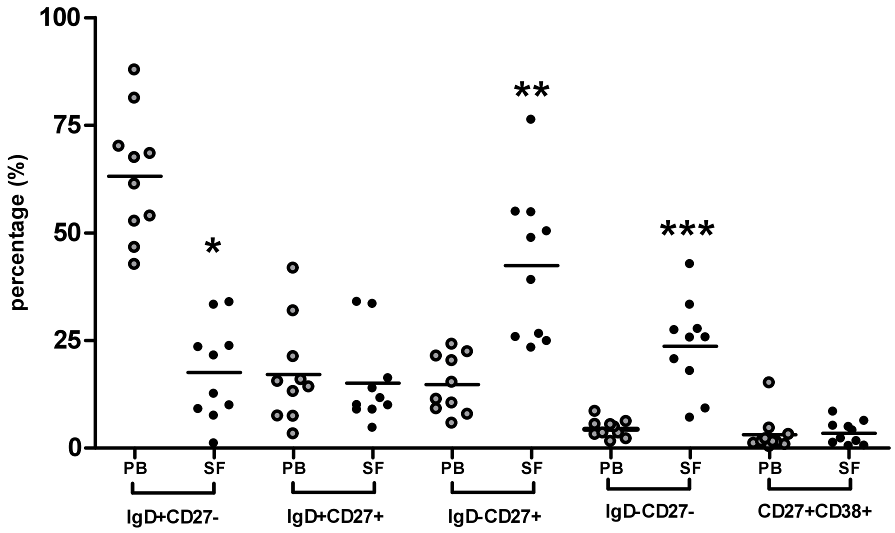

Supplementary Figure 2. FACS analysis of B cell subpopulations in paired RA PB and SF samples (n=10). $IgD^{+}CD27^{-}$ cells percentages in PB vs SF *$p<0.0001$; $IgD^{-}CD27^{+}$ cells percentages in PB vs SF **$p=0.002$ and $IgD^{-}CD27^{-}$ cells percentages in PB vs SF ***$p=0.01$; Wilcoxon test.

Supplementary Figure 3

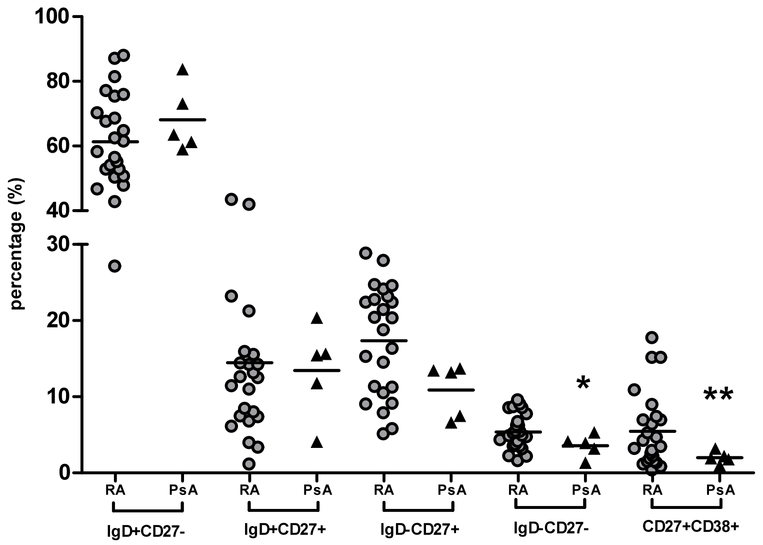

Supplementary Figure 3. FACS analysis of B cell subpopulations in PB of RA (n=24) and PsA (n=5) patients. $IgD^{-}CD27^{-}$ cells percentages in RA vs PsA patients *p=0.03; Mann-Whitney U-test.

Supplementary Figure 4

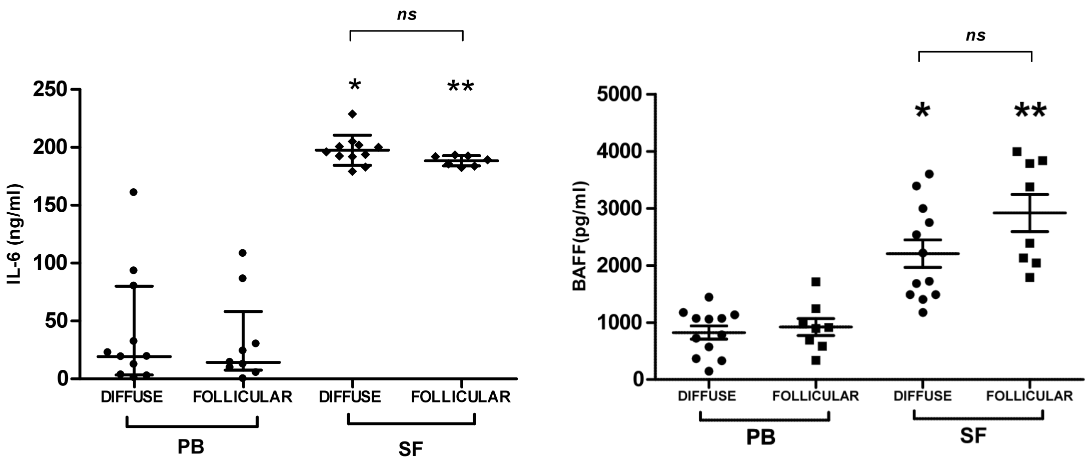

Supplementary Figure 4. IL-6 and BAFF levels in paired-PB and SF (n=20) samples of RA patients based on the synovial pattern; *p=0.001 and p=0.01 Diffuse SF vs Diffuse PB for IL-6 and BAFF levels respectively; **p=0.001 Follicular SF vs Follicular PB for IL-6 and BAFF levels; ns: not significant; Mann-Whitney U-test.

Supplementary Figure 5

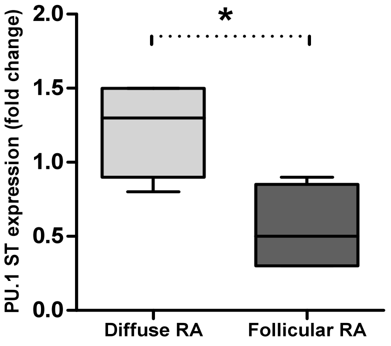

Supplementary Figure 5 . PU.1 expression in lysate of whole synovial tissue of RA patients with follicular (n=10) and diffuse (n=10) synovitis, *p=0.02 diffuse vs follicular synovitis; Mann-Whitney U-test. Fold-change calculated using mean value of RA patients with diffuse synovitis as a reference.

Supplementary Figure 6

a

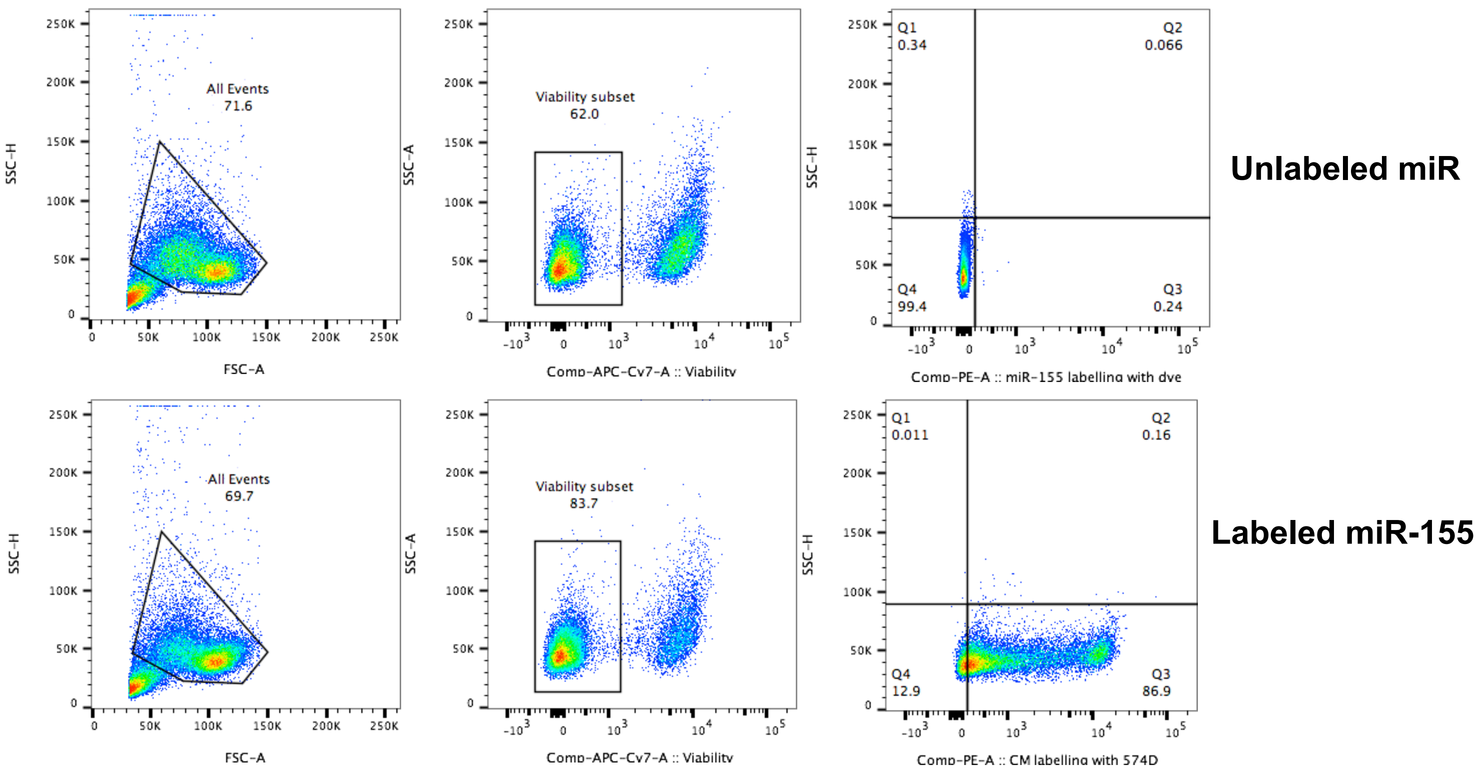

b

Exp number	Viability (%)	TE (%)
**Transcription Factors Experiments**		
**1**	75.3	67.1
**2**	83.7	86.9
**3**	43.9	62.2
**4**	76.6	78.3
**5**	73.1	64.2
**6**	75.6	74.9
**Total IgG Production Assay**		
**1**	67.6	98.4
**2**	45.5	90.4
**3**	89.9	86.7
**4**	89.6	86.9
**5**	91.1	88.9

Supplementary Figure 6a,b. (a) Representative dot plot of transfection efficiency of RA PB CD19^{+} cells with miR-155 inhibitor. **(b)** Summary of viability and transfection efficiency (TE) of PB CD19^{+} cells from RA patients transfected with miR-155 inhibitor (miR-155I).

Supplementary Table 1

a	Transcription Factors experiments	
	RA (n=6)	HC (n=5)
**Female, n(%)**	6 (100)	4 (80)
**Age, years (mean±SD)**	57.7 ± 14.6	43.6 ± 6.4
**Disease Duration, months (mean±SD)**	9.7 ± 3.9	-
**DAS28, (mean±SD)**	3.0 ± 1.4	-
**ACPA positivity, n(%)**	6 (100)	-
**ESR, mm/1st hour (mean±SD)**	11.0 ± 6.7	-
**CRP, mg/dL (mean±SD)**	7.7 ± 8.4	-

b	IgG production assay experiments
	RA (n=5)
**Female, n(%)**	5 (100)
**Age, years (mean±SD)**	58.4 ± 15.2
**Disease Duration, months (mean±SD)**	18.8 ± 8.4
**DAS28, (mean±SD)**	4.2 ± 1.1
**ACPA positivity, n(%)**	5 (100)
**ESR, mm/1st hour (mean±SD)**	24.0 ± 14.1
**CRP, mg/dL (mean±SD)**	12.6 ± 6.9

c	B cell subpopulations sorting experiments	
	RA (n=6)	HC (n=6)
**Female, n(%)**	5 (83.3)	5 (83.3)
**Age, years (mean±SD)**	58.3 ± 15.8	51.8 ± 14.8
**Disease Duration, months (mean±SD)**	17.5 ± 9.9	-
**DAS28, (mean±SD)**	4.4 ± 1.8	-
**ACPA positivity, n(%)**	6 (100)	-
**ESR, mm/1st hour (mean±SD)**	64.0 ± 39.0	-
**CRP, mg/dL (mean±SD)**	27.4 ± 30.1	-

Supplementary Table 1a,c. Demographical, clinical and immunological parameters of enrolled RA patients and healthy control subjects for evaluation of the impact of miR-155 on transcription factors **(a)**, antibody production **(b)** and distribution of miR-155 in B cell subsets **(c)**. **DAS**: Disease activity score; **ACPA**: Anti-citrullinated peptides antibodies; **ESR**: Erythrocyte sedimentation rate; **CRP**: C-reactive protein; **RA**: Rheumatoid arthritis. **SD**: standard deviation.
